# Enhancing the role of vaccines in combatting antimicrobial resistance

**DOI:** 10.1016/j.vaccine.2017.09.053

**Published:** 2017-12-04

**Authors:** Charles Clift, David M. Salisbury

**Affiliations:** Centre on Global Health Security, Chatham House, 10 St James’s Square, London SW1Y 4LE, United Kingdom

**Keywords:** Vaccines, Antimicrobial resistance, Antibiotics, Valuation

## Abstract

Interest in addressing antimicrobial resistance (AMR) has grown recently but little effort has been made to consider how existing and new vaccines could impact on AMR. A 2017 Chatham House meeting considered the role of vaccines and how to demonstrate their value through their impact on AMR. Ways existing vaccines have reduced antibiotic prescribing and the prevalence of some resistant organisms were reviewed. Other new vaccines could have a similar impact. In gonorrhoea, where complete resistance has developed, vaccine may be the best option. Valuing the impact of vaccines on AMR was challenging: there were difficult methodological issues and a lack of data for modelling. A participant poll suggested priorities for accelerated vaccine development were tuberculosis, typhoid, influenza, RSV and gonorrhoea. More evidence is needed to convince policymakers but that vaccine development projects should be considered by funders on the same basis as those for new antibiotics or diagnostics.

## 1 Introduction

As the political interest in addressing the growing threat from antimicrobial resistance (AMR) has intensified, much attention has been focussed on ways in which new incentives for research and development (R&D) can be provided to hasten the development of new antimicrobials which can attack pathogens which have become resistant, or where resistance is growing. It has been estimated that about $500 million in new funding had been allocated in 2016 from 13 existing or new initiatives whose principal purpose is to accelerate the development of new antibiotics [1].

There is no doubt that addressing a threat as significant and complex as AMR requires a portfolio of solutions – including new antimicrobials, better diagnostics and better stewardship. It also requires that more attention be paid to a range of measures that prevent infection and reduce the use of antimicrobials including improved sanitation and the wider use of vaccines. For diseases where AMR has become rampant, vaccines may be the best or only way to save lives.

While the role of vaccines in combatting antimicrobial resistance has been mentioned or considered in recent reports and plans on AMR, much less effort has been put into supporting the greater use of existing vaccines and the development of new ones to address AMR. An exception was the UK’s 2016 Review on Antimicrobial Resistance (chaired by Lord O’Neill) which argued in its main report [2], and in a background paper on vaccines and alternative approaches [3], that vaccines, as a means to combat AMR, were under-researched and deserved a greater level of investment. The report noted that spending on vaccine R&D lags behind that on new drugs, and in the current global healthcare paradigm, far more effort and reward goes to treatment than to prevention.

## 2 Discussion

To investigate the potential for vaccines further, the Centre on Global Health Security at Chatham House convened a meeting in London in March this year of vaccine experts and stakeholders, including international and regional organizations, economists, modellers and scientists from several vaccine-producing companies. The purpose of the meeting was to review current knowledge and activities on the role of vaccines in combatting AMR, and to consider the issues involved in modelling how their value in this role could be established. This was with a view, in particular, to ensuring that national and international policies on their use and support for R&D efforts properly recognized the contribution of vaccines in mitigating the growth of AMR [4].

The general case for using existing vaccines more widely and developing new ones to combat AMR is quite easy to make in principle. The development of AMR is inherent in the use of antibiotics. No new classes of antibiotic have been discovered in the last 25 years and AMR continues to grow. Even if recent initiatives are successful in stimulating the development of new classes of antibiotic, they will most likely encounter resistance. In gonorrhoea, resistance has developed to each successive antibiotic used to the extent that we are now nearly at the stage where it is becoming untreatable. Antibiotic development is therefore comparable to the work of Sisyphus, continually rolling his boulder up to the top of the hill only to see it roll down again. Because AMR is intrinsic to antibiotic use, the only long term solution to AMR is preventing the infections that necessitate their use. There are many factors that contribute to prevention, such as improved living standards and water and sanitation, but vaccines have been a key element in disease prevention globally for almost two centuries. Vaccines should therefore be considered as key weapons in the fight against AMR.

The meeting provided some powerful examples where existing vaccines have reduced antibiotic use. The pneumococcal conjugate vaccine has reduced overall antibiotic prescribing and also the prevalence of resistant organisms [5], [6]. Globally, the influenza season accounts for a significant proportion of antibiotic use. Vaccination not only prevents the widespread inappropriate primary use of antibiotics [7] but also their use for secondary bacterial infections [8]. A respiratory syncytial virus (RSV) vaccine, now in development, could have a huge impact on antibiotic use and AMR. A vaccine for Group B streptococcus (GBS) could avert many infections in mothers and infants, and in reducing AMR. It would also avoid the increasing prophylactic use of antibiotics in pregnant women screened for GBS carriage or considered at risk. According to one study in Nepal, resistance to fluoroquinolones was encountered in over 80% of typhoid infections [9]. In gonorrhoea, in the absence of any promising new antibiotic treatments in the pipeline, vaccination may be the only option although there are also many challenges in developing a vaccine. However, the unexpected impact of group B meningococcal vaccination in New Zealand leading to fewer cases of gonorrhoea in vaccinated cohorts provides some encouragement [10].

However, there are many factors, including finance, that inhibit the wider use of existing vaccines (e.g. global coverage of PCV vaccines is only 37%) and there are scientific as well as commercial challenges in developing several potential new vaccines (e.g. for gonorrhoea, tuberculosis, HIV) which would impact AMR. However, convincing decision makers in ministries of health and finance as well as in the scientific community and donors, to invest more in vaccine distribution and development requires hard evidence of cost-effectiveness. The meeting grappled with the problem of how a value could be put on the contribution of vaccines to combat AMR.

Three scenarios can be envisaged for modelling the impact. First, there is the direct impact of vaccination on reducing antibiotic use through fewer cases of infection translating into fewer antibiotics used and therefore fewer opportunities for resistance to occur. But the AMR impact for any individual vaccine may be fairly small compared to the ‘conventional’ health and economic benefits associated with reducing illness or death. For example, a flu vaccine by itself might not reduce inappropriate antibiotic use when other viral infections could be the cause of flu-like symptoms, demonstrating the need for reliable, fast and cheap point of care diagnostics to make the most of vaccines in reducing AMR. Secondly, there is the ‘herd effect’ of vaccines that reduces infections in the non-vaccinated thereby reducing antibiotic use. Again, the benefits attributable to AMR reduction may be relatively small. The third scenario is when there are no effective antibiotics for a particular disease (as is nearly the case with gonorrhoea). Because the health and economic costs of gonorrhoea are small relative to some other diseases (e.g. infertility rather than death), and the challenges of vaccine development are considerable, decision makers may not be swayed. At the extreme, there could be a level of AMR that makes a wide range of surgery and treatments extremely risky in the absence of antibiotics e.g. bone marrow transplants, chemotherapy, and bowel and orthopaedic surgery. Because the potential health and economic costs of the latter are potentially astronomic, this could make the case for vaccines seem unanswerable, but decision makers will not take action unless convinced that the risk is (very) high and there are optimistic prospects for new vaccine development. The meeting considered that modelling the impact of vaccines on AMR was complex and more work was needed both in clarifying methodologies for modelling the AMR impact of vaccines and in generating more data to populate the models. Extensive assumptions would be needed to link the impact of vaccines in preventing infections to the reductions of antimicrobial use that would result and the latter’s impact on the development of resistance.

As an exercise in prioritisation participants at the meeting were invited to consider what vaccines would have the greatest impact on AMR. The top five organisms chosen for accelerated vaccine development were tuberculosis, typhoid, influenza, RSV and gonorrhoea. This choice is significantly different to the prioritisation undertaken recently by the World Health Organization of infections for which new antibiotics are needed [11]. It seems that participants prioritised vaccines for investment based on their perceptions of the burden of disease, the current prevalence of AMR, the feasibility of developing new vaccines and the extent to which investments were already being made for commercial reasons (e.g. pneumococcal vaccines).

Several argued that vaccination to reduce the use of antibiotics in animals could be as important as, if not more important than, in humans. More than 70 percent of the antibiotics deemed medically important for human health by the FDA sold in the United States (and over 50 percent in most countries in the world) are used in livestock [12].

## 3 Conclusions

Policy makers at national and international level need to be presented with more evidence, underpinned by economic modelling, on the value of vaccines in combatting AMR so that the latter’s development and use are encouraged and supported. Those responsible for vaccine research, international organisations that support vaccine research and vaccination, and national governments need to be persuaded that investment in vaccines will play a significant role in the reduction of AMR. Researchers and manufacturers need to be offered appropriate incentives, in particular for vaccines that could have a high impact on AMR but where the commercial market prospects are uncertain. At the very least new and potential future AMR initiatives should consider vaccine projects on the same basis as proposals for new antibiotics or diagnostics. There should be regular reviews of progress in vaccine development and promotion such that the ‘vaccine AMR value’ concept is prominent whenever strategies for combatting AMR are being considered. And there needs to be a similar initiative in livestock production, to consider measures necessary to reduce antibiotic use, including enhanced use of vaccination.
